# Surgical Stress Promotes Tumor Progression: A Focus on the Impact of the Immune Response

**DOI:** 10.3390/jcm9124096

**Published:** 2020-12-18

**Authors:** Amblessed E. Onuma, Hongji Zhang, Lindsay Gil, Hai Huang, Allan Tsung

**Affiliations:** Division of Surgical Oncology, Department of Surgery, The Ohio State University Wexner Medical Center, Columbus, OH 43210, USA; amblessed.onuma@osumc.edu (A.E.O.); hongji.zhang@osumc.edu (H.Z.); lindsay.gil@osumc.edu (L.G.); hai.huang@osumc.edu (H.H.)

**Keywords:** surgical stress, microenvironment, metastasis, neutrophil extracellular traps, inflammation

## Abstract

Despite advances in systemic therapies, surgery is crucial for the management of solid malignancy. There is increasing evidence suggesting that the body’s response to surgical stress resulting from tumor resection has direct effects on tumor cells or can alter the tumor microenvironment. Surgery can lead to the activation of early and key components of the innate and adaptative immune systems. Platelet activation and the subsequent pro-coagulation state can accelerate the growth of micrometastases. Neutrophil extracellular traps (NETs), an extracellular network of DNA released by neutrophils in response to inflammation, promote the adhesion of circulating tumor cells and the growth of existing micrometastatic disease. In addition, the immune response following cancer surgery can modulate the tumor immune microenvironment by promoting an immunosuppressive state leading to impaired recruitment of natural killer (NK) cells and regulatory T cells (Tregs). In this review, we will summarize the current understanding of mechanisms of tumor progression secondary to surgical stress. Furthermore, we will describe emerging and novel peri-operative solutions to decrease pro-tumorigenic effects from surgery.

## 1. Introduction

Treatment of solid malignancy is complex and often varies based on the type of tumor. Advances in systemic treatment with chemotherapy or immunotherapy have led to improved progression-free survival and overall survival in some solid tumors [1,2,3,4]. However, in the majority of solid tumors, treatment with systemic therapy is not curative and surgery remains the only chance for cure. Curative-intent surgery, when appropriate, has been shown to improve patient survival. Surgeons have long speculated that oncologic resection, a necessary step in the treatment of solid tumors, can also facilitate tumor outgrowth and the metastatic process. There is mounting evidence from in vivo experiments that surgery-induced stress is a significant factor in promoting tumor growth and metastasis [5,6,7,8,9,10].

Surgery elicits a systemic response to injury that leads to alterations in the immune and hematologic systems. For example, surgery promotes fibrin and platelet clot formation, one of the first responses activated by the body after injury. Activated platelets have been shown to coat tumor cells and protect them for detection and clearance by NK cells [6,11]. These tumor cells coated by platelets are sometimes referred to as circulating tumor cells. The formation of thrombin and fibrin clots can also promote a postoperative hypercoagulable state that has been implicated in promoting metastatic disease. In addition, immunosuppression induced by surgical operation either by laparotomy or laparoscopy can result in accelerated tumor growth and hematogenous metastasis [12]. In this review, we will cover underlying mechanisms of tumor progression secondary to responses in the body induced by surgery. First, we will focus on how coagulation components involved in local tissue injury can promote distant metastasis. We will also discuss how surgery can create an immunosuppressive state that favors recurrence and metastasis. Finally, we will describe proposed solutions to mitigate this side effect of surgical stress.

## 2. Platelet Activation and Tumor Development

Platelets are small, anucleate cells found in circulation, measuring 2–5 μm in diameter with a thickness of 0.5 μm [13,14]. The formation of platelets from megakaryocytes is a well-regulated process. Following their formation, platelets exist in circulation for 5–7 days where they primarily function as regulators of hemostasis and thrombosis [13]. However, platelets can also be activated during inflammatory conditions, leading to interactions between leukocytes and endothelial cells. Furthermore, there is evidence that platelets play a role in immunity and in modulating pathophysiological responses during inflammation [15]. Once activated, platelets quickly interact with innate immune cells and exert immunomodulatory effects directly through cell–cell contact or remotely via the release of chemokines and cytokines from neutrophils or monocytes [16,17]. Activated platelets directly interact with leukocytes via P-selectin (CD62P) binding with P-selectin glycoprotein ligand 1 (PSGL-1). This interaction between platelets and leukocytes results in leukocyte recruitment, activation, extravasation, phenotype switch and change in effector functions [17]. Platelets mediate leukocyte rolling through glycoprotein 1b (GP1b) and GPIIb/IIIa [18,19,20] and regulate monocyte function by modulating their activation, polarization and differentiation [21].

It is widely known that millions of cells are shed from solid tumors into the circulation daily; however, few clinically metastatic foci are formed. During tissue injury caused by surgery, the coagulation pathway including platelets is activated to begin the process of wound healing [22]. This process has also been implicated in protecting circulating tumor cells. Platelets attach to tumor cells through adhesion receptors P-selectin, GP1b-IX-V and GPIIb/IIIa, of which GPIIb/IIIa plays a major role in the process of tumor and platelet interaction [23,24,25,26]. GPIIb/IIIa has been shown to interact with tumor cell receptor α_v_β_3_ to promote tumor cell-platelet aggregation. This aggregate can promote the arrest of tumor cells to blood vessel walls, thereby preventing their destruction by shear forces generated by blood flow [27]. Surgery also promotes the release of pro-inflammatory cytokines (IL-1, IL-6, TNF), which have been shown to increase the production of fibrinogen [28]. Fibrin and platelet clots induced by surgery can coat the tumor cells, protecting them from detection and attack by NK cells [6,11].

Tumor cells can induce platelet activation to promote their progression through numerous mechanisms. The secretion of thromboxane A2 (TXA_2_) and Adenosine Diphosphate (ADP) from cancer cells can further augment platelet activation and aggregation and promote tumor cell survival within the circulation [25,27,29,30,31]. Similarly, tumor cells shed small extracellular vesicles referred to as exosomes. Exosomes contain proteins, lipids and nucleic acids that are important for cancer cell communication and aid in the proliferation and survival of tumor cells [32]. These expose the transmembrane tissue factor that initiates the extrinsic coagulation cascade, resulting in thrombin generation and amplifying platelet activation and aggregation [33,34,35]. The upregulation of tissue factor expression has been linked with a hypercoagulable state and enhanced tumor cell metastasis [36]. The release high-mobility group box1 (HMGB1) protein from tumors can also promote cancer progression [37]. HMGB1 then binds to platelets via toll-like receptor 4 (TLR4) to mediate cell adhesion and enables tumor cell survival and metastasis.

A study by Seth et al. was one of the first experimental studies to demonstrate that surgery promotes cancer metastasis through a coagulation-dependent mechanism [6]. Using a murine model, surgical stress was induced in immune-competent Balb/c mice by left hepatectomy or left nephrectomy before intravenous inoculation with colon cancer cell line CT26LacZ to establish pulmonary metastasis. The total number of gross metastasis on the left lobe as well as quantification of tumor cells on hematoxylin and eosin (H & E) staining were used to characterize tumor metastasis. The authors found that surgical stress resulted in increased metastases, while different anticoagulants and platelet depleting agents attenuated this effect. Finally, this increase in tumor cell emboli survival secondary to surgical stress was eliminated by NK cell depletion. Their work suggests that surgical stress promotes the formation of fibrin and platelet clots around tumor cell emboli, impairing NK cell-mediated tumor cell clearance, and perioperative anticoagulation diminishes this effect. By using Galphaq-deficient mice, a protein necessary for platelet activation, Palumbo et al. demonstrated that platelet activation contributed to tumor progression [11]. Furthermore, they found that platelets and fibrinogen promote metastasis in part by hindering intravascular tumor clearance by NK cells.

Surgical stress may promote tumor metastasis through another mechanism termed immunothrombosis. Immunothrombosis is the process that ensues from the interaction between neutrophil extracellular traps (NETs) and the coagulation system [38,39]. NETs and their role in tumor progression are further described in Section 3 below. Our group has shown that NETs can exacerbate distal organ injury (in the lung and kidney) after liver surgery through activation of systemic procoagulant state and diffuse microvascular immune thrombi [40]. Platelet microparticles can promote tumor growth due to their ability to transport multiple functional factors and membrane receptors associated with tumor growth and proliferation [41]. These microparticles express tissue factor (TF), which triggers the creation of thrombin and activation of proteinase-activated receptor 1(PAR-1), leading to vascular endothelial growth factor (VEGF) secretion and enhancement of angiogenesis [42,43].

## 3. Neutrophils and Neutrophil Extracellular Traps

Neutrophils are the most abundant type of leukocytes in humans, accounting for approximately 70% of all leukocytes. They are an important part of the innate system that protects the host from pathogens. These first-line responders also play an important role in linking inflammation to cancer progression [44,45]. The influx of neutrophils after surgery has been shown to promote tumor capture and growth, and our work has previously shown that liver metastatic burden is significantly increased following surgical stress [46,47].

**Figure 1:**
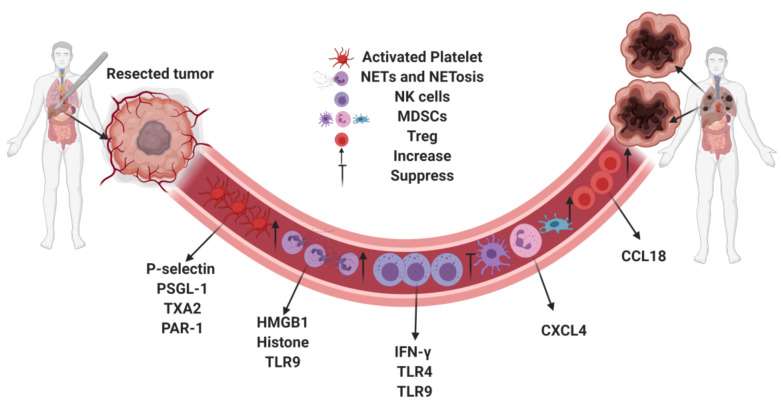
Surgical stress promotes tumor metastasis by regulating the innate and adaptive immune systems via mediators.

Neutrophils can react to tissue injury by forming neutrophil extracellular traps (NETs) through a process termed NETosis [48]. NETs consist of expelled DNA, which are decorated in histones and granular proteases such as neutrophil elastase (NE) and myeloperoxidase (MPO) [49]. NETs were originally discovered to play a role in a neutrophil’s defense strategies against microbes [50]. However, recent discovery has shown that NETs can also play a role in tumor progression. NETs sequester circulating tumor cells from circulation and promote tumor cell invasion and metastasis [51,52]. Our previous study found that in a cohort of patients undergoing curative-intent liver resection for metastatic colorectal cancer, increased NET formation was correlated with worse outcomes, specifically a 4-fold decrease in disease-free survival [47]. Likewise, in a murine model of surgical stress utilizing ischemia-reperfusion, we found an increase in NET formation was associated with increased development and progression of metastatic colorectal cancer. This effect was diminished by NET inhibition either through local treatment with deoxyribonuclease (DNase) or inhibition of peptidylarginine deaminase (PAD4), an essential protein for NET formation [47]. NETs can also trigger HMGB1 release. As outlined in the previous section, not only does HMGB1 bind platelets via TLR4, it also activates TLR9-dependent pathways in cancer cells, thereby promoting tumor cell proliferation, adhesion, migration and invasion after surgical stress. In addition, NETs have been implicated in awakening dormant tumor cells after inflammation [53]. Figure 1 summarizes the effect of surgical stress on the innate and adaptive immune responses with their corresponding mediators.

## 4. Immunosuppression

Tumors often create an immunosuppressive microenvironment to help them escape immune surveillance, thereby favoring tumor progression and metastasis [54,55]. The inflammatory response following surgery can promote a similar systemic immunosuppressive state [56,57]. In a study investigating if surgery drives the development of tumor immunosuppression in recurrent tumors, Predina et al. hypothesized that surgical stress in the form of “surgical wounding” may generate an inflammatory response in the residual tumor [57]. Utilizing an experimental model of lung carcinoma, they harvested tumors at various time points, and lysates were analyzed for cytokine changes. The authors found that there were significant increases in the various pro-tumorigenic cytokines (VEGF, IL-1β, IL-6, IL-10, MCP-1, and TGF-β) with a significant decline in IFN-γ after surgical resection. Additionally, tumors that had undergone partial tumor resections were found to have alternatively activated macrophages and T regulatory cells that prevented CD8+ T cell recruitment to the tumors and led to faster recurrence. This work demonstrates that surgery can produce immunosuppressive cellular changes responsible for rapid recurrent tumor growth [57]. Previous studies have also demonstrated a global decrease in cytolytic CD8+ T cells after surgery [58,59]. In a mouse model of melanoma, the inflammatory response following surgery led to cancer progression secondary to functional impairment of tumor specific CD8+ T cells [60]. Major surgery, modeled by laparotomy and nephrectomy, resulted in significant decrease in IFN-γ, TNF-α, and Granzyme B-secreting splenic CD8^+^ T cell starting at after surgery and lasting 7–10 days [60].

NK cells, a main source of IFN-γ, are cytotoxic lymphocytes that play an important role in cancer immunosurveillance and infection. Clinical studies have correlated intratumoral NK cell density with cancer prognosis and incidence [61,62,63]. NK cell IFN-γ secretion in peripheral blood obtained from patients who underwent colorectal surgery has been assessed preoperatively and postoperatively [64]. The authors found that IFN-γ secretion was severely suppressed following cancer surgery with more than 90% of patients being below the detection levels on post-operative day one and lasting up to two months [64]. This IFN-γ suppression was present regardless of gender, age or cancer stage. There was no difference in IFN-γ suppression based on the surgical approach when comparing open versus laparoscopic surgery. It is important to note that the absolute number and subsets of NK cells did not differ following surgery, confirming that surgical stress affects the function of NK cell and not the population of cells. The authors of this study did not comment on tumor recurrence or long-term outcomes based on IFN-γ suppression level; however, other studies have associated transient dysfunction of NK cell cytotoxicity after surgery with cancer recurrence and metastasis in both animal and human studies [5,64,65,66].

Another mechanism by which post-surgical systemic immune changes leads to an immunosuppressive environment is through the downregulation of the chemokine (C-X-C motif) ligand 4 (CXCL4) and recruitment of intratumoral-myeloid-derived suppressor cells (MDSC). Using a syngeneic transplantation tumor model of murine CT26 colon cancer, Xu et al. found that laparotomy promoted in vivo tumor growth and angiogenesis [67]. First, they found that CXCL4 expression was significantly decreased following laparotomy compared to sham. Additionally, they found that overexpressing CXCL4 in these CT26 cells led to significant decrease in tumor growth induced by laparotomy. IHC staining for VEGF and CD31 in these CXCL4 overexpressed tumors revealed that angiogenesis was attenuated in the laparotomy group. Finally, they investigated whether CXCL4 regulates the recruitment of MDSCs into tumor tissue and found that CXCL4 overexpression decreased MDSC migration in vitro, and flow cytometry analysis revealed that CD11b^+^/Gr1^+^ MDSC cells were upregulated in the tumor tissues and peritoneal cavity after laparotomy. Using clinical data, the authors found that the expression of CXCL4 and MDSC percentage were negatively correlated and both were linked with overall survival [67].

**Figure 2:**
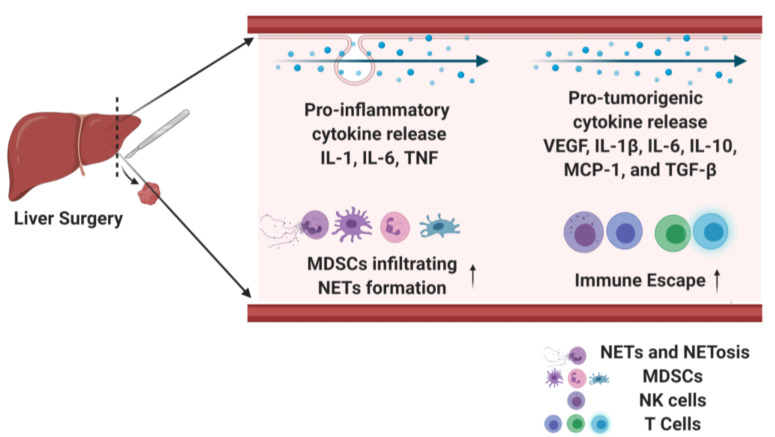
Surgical stress leads to pro-inflammatory and pro-tumorigenic cytokine release, which increase tumor metastasis.

Surgical stress has been shown to contribute to colon cancer progression by increasing cytokine levels of the C-C Motif Chemokine ligand 18 (CCL18) and T regulatory cell recruitment [68]. A surgical laparotomy on CT26 tumor-bearing mice results in the upregulation of CCL18. The presence of CCL18 was associated with the subsequent increased recruitment of Tregs in the tumor, peritoneal cavity and peripheral blood. CCL18 knockdown significantly reduced tumor growth and angiogenesis [68]. These findings were confirmed in human samples where serum CCL18 level was positively correlated with Treg populations in colon cancer patients. Figure 2 summarizes major cytokines that are released after surgical stress, promoting tumor metastasis.

## 5. Countering the Effects of Surgical Stress on Tumor Progression

Numerous studies have now shown that the immune response following surgery can promote tumor progression. This perioperative window is an understudied time period where novel therapeutics can abrogate the effects of surgical stress on tumor progression. To address pro-tumorigenic coagulation changes that occur during surgery, hydroxyethyl starch (HES) has been shown to decrease circulating tumor cells in patients undergoing colorectal cancer resection through the inhibition of platelet activation [69]. The use of other anticoagulation agents to prevent tumor progression has been studied extensively and shown to improve survival in cancer patients [6,70]. The use of low-molecular-weight heparin, dalteparin, has been shown to cause a statistically significant improvement in overall survival relative to oral anticoagulant therapy in patients with solid tumors who were not known to have metastatic disease at the time of the thromboembolic event [71]. Perioperative administration of either five different anticoagulants (tinzaparin, dalteparin, hirudin, warfarin and platelet depletion with α-platelet GPIbα) resulted in significant attenuation of pulmonary metastases after surgical stress in a murine model [6]. There is currently a multicenter randomized controlled trial (PERIO-01, NCT01455831) looking at the extended use of perioperative low-molecular-weight heparin (tinzaparin) to improve cancer specific survival follow surgical resection of colorectal cancer [72]. Our group is also currently investigating the benefits of NET inhibition by administering DNase to decrease the capture of circulating tumor cells after surgical stress in hepatic metastatic colorectal cancer [46].

Currently, there is a phase 1 clinical trial (PERIOP-04, NCT02998736) looking to determine if Tadalafil, a phosphodiesterase inhibitor, in combination with the influenza vaccine can decrease the chances for metastasis post-surgery for patients with abdominal cancer undergoing resection [73]. The rationale behind this clinical trial is based on two key studies [74,75]. In the first study, the authors found that perioperative administration of phosphodiesterase inhibitor reduces surgery-derived granulocytic-MDSC function via downregulation of arginase 1, IL4Ra and reactive oxygen species, enhancing NK cell cytotoxicity and decreasing postoperative disease recurrence [75]. In the second study by the same authors, they found that preoperative influenza vaccination prevents postoperative NK-cell dysfunction, abrogating tumor metastasis in murine models and increasing the activation of NK cells in cancer patients [74].

The use of beta-blockers or cyclooxygenase (COX) inhibitors during the perioperative period has been investigated and has been shown to suppress molecular pathways involved in metastatic progression [76]. These medications work by inhibiting pro-tumorigenic mediators, preserving cytokines that enhance the activity of NK cells and cytotoxic T cells, decreasing intra-tumoral monocytes, and decreasing the activity of immune-suppressive CD4+ T cells [76,77]. Surgical stress induced by surgically wounding tumor-bearing mice at a distant anatomical site has been correlated with a significant increase in the outgrowth of murine mammary carcinoma cells [78]. This work is important as it elucidates that inflammation secondary to surgical stress can promote tumor development, and this inflammation does not need to originate from surgical resection. Biopsies or elective surgical operations for other purposes apart from the primary tumor can also influence the primary tumor growth and metastasis. Perioperative treatment with nonsteroidal anti-inflammatory drugs significantly inhibited the impact of surgical stress on tumor growth. Although the long-term impact of these treatments on tumor progression have yet to be fully assessed, the association between the use of perioperative beta-blockers and COX-2 inhibitors and progression-free survival in breast cancer patients has been studied [79,80]. A subset of cancer patients may eventually be identified who are likely to benefit from concomitant blockade of stress physiology and more traditional anti-tumoral therapy.

Recent developments in immunotherapy may play an additional role in reducing surgical-stress-induced tumor progression. Toll-like receptor 4 (TLR4) and TLR9 agonists have been shown to decrease cancer metastasis by increasing NK cell cytotoxicity during the perioperative period [81,82]. The use of tumor vaccines in the perioperative period has also been investigated as a potential therapeutic target to prevent tumor progression. Given the suppressive effect of surgery on NK cell function, Tai et al. found that perioperative administration of oncolytic parapoxvirus ovis (ORFV) and vaccinia virus can reverse NK cell suppression, which correlates with a reduction in the postoperative formation of cancer metastases [83]. There are clinical trials that have studied preoperative administration of INF-alpha or IL-2 therapy that have been shown to prolong overall survival in patients with various solid malignancies [84,85,86]. In a non-randomized Phase II trial of patients with renal cell carcinoma, 60 patients received IL-2 preoperatively prior to nephrectomy compared to the control group [84]. The authors found that the patients who received IL-2 preoperatively had a significantly higher 5-year disease free survival (DFS) than the control group. These immunotherapies were well tolerated with minimal adverse events [85].

## 6. Concluding Remarks

Despite the advancement in non-surgical therapies for malignancies, surgical resection remains a cornerstone treatment modality for solid tumors. However, evidence has shown that the unintended effect of the body’s inflammatory and immune responses following surgery for cancer can result in a pro-tumorigenic environment. Understanding the mechanisms underlying this effect in the perioperative period will help to identify solutions to improve cancer prognosis for solid malignancies.
